# Long-term reproducibility and clinical utility of endometrial receptivity analysis in guiding personalized embryo transfer: case reports of sustained success over four years post-endometrial biopsy

**DOI:** 10.3389/frph.2026.1769800

**Published:** 2026-02-06

**Authors:** Ya-Jun Dong, Yan Huang, Shu-Hong Luo, Hong-Xia Ye, Yan Jia

**Affiliations:** Department of Reproductive Immunology, Sichuan Jinxin Xi'nan Women’s and Children’s Hospital, Chengdu, Sichuan, China

**Keywords:** case reports, endometrial receptivity analysis, personalizedembryo transfer (pET), recurrent implantation failure (RIF), window of implantation (WOI)

## Abstract

**Aims:**

To demonstrate the long-term stability and clinical utility of endometrial receptivity analysis (ERA) test in guiding personalized embryo transfer (pET) for patients with a history of recurrent implantation failure (RIF), even after an intervening live birth.

**Methods:**

Two RIF patients, who had previously achieved a live birth via ERA-guided pET, sought a second pregnancy. For their subsequent personalized frozen-thawed embryo transfer cycles, progesterone administration timing was adjusted based on the receptive window identified by a single ERA test conducted more than four years earlier. No repeat endometrial biopsy was performed in either patient.

**Results:**

Both patients underwent pET following the recommendations derived from their original ERA results. Both achieved successful clinical pregnancies and subsequently delivered healthy live infants.

**Conclusion:**

These findings preliminarily suggest that repeat endometrial biopsy might not be necessary for subsequent pET in RIF patients with similar clinical backgrounds. However, given the small sample size of this case series, further large prospective studies are still needed to confirm the long-term utility of initial ERA results and the appropriateness of omitting repeat biopsies.

## 1 Introduction

Recurrent implantation failure (RIF) remains a significant challenge in the field of assisted reproductive technology (ART), causing frustration for both patients and clinicians alike. The etiology of RIF is multifactorial, involving both embryonic and maternal factors (1, 2). Endometrial receptivity (ER) contributing to embryo-endometrial synchrony is considered as a primary cause for implantation failure (3). The window of implantation (WOI), that fleeting period when the endometrium is most receptive to embryo attachment, holds the key to successful implantation (4). In recent years, a variety of diagnostic tools have emerged with the aim of identifying the personalized optimal timing for embryo transfer (5). The Endometrial Receptivity Analysis (ERA) test, which relies on the transcriptomic signature of over 200 genes, has established itself as a commercial tool for WOI identification (6). Many previous studies have reported its clinical efficiency in improving the pregnancy outcome especially in RIF patients (6–8). However, as a diagnostic test, the reproductivity data of it is still limited up to now (9). Whether a repeat endometrial biopsy for ER test is needed after a period for the induvial patients is not fully understood.

The following two case reports present experiences of patients with recurrent implantation failure who achieved pregnancy success by following the recommendations of the ERA test performed more than 4 years ago. These cases may offer insights into the long-term reproducibility and effectiveness of the ERA test in guiding personalized embryo transfer, extending the understanding of such tests' performance over extended periods following the initial biopsy and subsequent livebirth.

## 2 Case presentation

### 2.1 Case 1

#### 2.1.1 Patient history

The woman, born in 1988 and of Yi nationality, sought treatment at our hospital in April 2018 due to a seven-year history of infertility without contraception. In 2013, she underwent hysterosalpingography at an external hospital, which suggested possible bilateral tubal occlusion at the interstitial segment, but this issue was not addressed further. By August 2015, due to tubal factor infertility, she had undergone two cycles of *in vitro* fertilization (IVF) at another hospital, resulting in three embryo transfers, all of which failed to establish a pregnancy. From June to December 2019, she attempted two additional embryo transfer cycles at our hospital. The first cycle involved two D3 embryos (Grades 830 and Fusion II), and the second cycle transferred one D6 blastocyst (Grade 6B-B). Unfortunately, these transfers also did not result in successful pregnancies (Supplementary Tables S1 and S2).

#### 2.1.2 ERA test findings

In March 2020, due to the diagnosis of RIF with 5 transfer failures, she underwent hysteroscopy examination and an ERA test in an artificial cycle. The hysteroscopy showed no abnormalities. The ERA test report indicated that her endometrium was early-receptive, suggesting that her window of implantation (WOI) was delayed by 12 h, occurring 133 ± 3 h post-endometrial biopsy. Based on these findings, personalized embryo transfer (pET) for day-5/6 blastocysts should occur at 133 ± 3 h after progesterone administration, or for day-3 embryos at 85 ± 3 h.

#### 2.1.3 Treatment and outcomes

Despite adhering to the report-guided timing, three subsequent embryo transfers from April to October 2020 with remaining frozen embryos were unsuccessful. Undeterred, the patient pursued further IVF treatment, which resulted in five new frozen embryos. In March 2021, following the ER test recommendations, a blastocyst was transferred 5.5 days after initiating progesterone administration. Notably, the method of progesterone delivery was changed from intramuscular injections to vaginal capsules due to the patient's inconvenience with injections and her request for an alternative. Following the embryo transfer, she successfully conceived, ultimately resulting in the birth of a healthy, full-term baby girl via cesarean section in December 2021 (Supplementary Table S2).

As of June 2024, the couple planned to have a second child and requested to proceed with thawed embryo transfer again. In September 2024, a grade 6BB blastocyst (originated from one of the 4BB blastocysts frozen in 2020) was transferred at the optimal transfer time recommended by the established ERA report, leading to another successful clinical pregnancy and delivered a baby boy. Remarkably, four and a half years after her last biopsy in March 2020, she has once again achieved a successful pregnancy through pET, even after delivering a child during the intervening period. By reviewing the past, the patient's menstrual cycles remained regular and stable throughout the 4-year period, with no clinically significant changes.

The quality of all blastocysts was assessed using the Gardner grading system, a validated standard that links morphological scores to implantation potential (10).

#### 2.1.4 Chronic endometritis screening

In March 2020, concurrent with the ERA biopsy, a hysteroscopy revealed a normal uterine cavity and smooth endometrium, with the pathology report indicating a late proliferative to early secretory phase endometrium without signs of inflammation. A subsequent hysteroscopy in July 2024, prior to her second successful pregnancy, also showed a normal uterine cavity.

#### 2.1.5 The protocol of ERA test

The ERA test was performed in a hormonal replacement therapy (HRT) cycle. Initially, Femerton® tablets (2 mg, orally, twice daily) and Estradiol Gel (5 g, externally, twice daily) were administered for a period. When the endometrial thickness is >7 mm and serum progesterone level is <1 ng/mL (3.18 nmol/L), progesterone administration was initiated at a dose of 60 mg intramuscularly once daily, along with two oral yellow Femerton® tablets twice daily, for a total duration of five days. On the fifth day of progesterone administration (*P* + 5), an endometrial biopsy was performed using a Pipelle catheter (Gynetics) to collect a sample size of approximately 50–70 mg from the uterine cavity. The collected tissue was promptly sent to Hangzhou Yizhen Medicine Laboratory in Hangzhou, China. The receptivity status of the tissue was determined using the HerRecepta platform. Briefly, RNA was extracted from the endometrial tissue and RNA sequencing was performed after RNA library construction. Based on the expression data of more than 240 genes, the final results were predicted based on inhouse predictor developed by Hangzhou HerAnova Biotechnology Co., LTD using python sklearn model. The receptivity results were reported as receptive, pre-receptive, early receptive, post-receptive and late receptive. The optimal WOI was also recommended in the test reports.

### 2.2 Case 2

#### 2.2.1 Patient history

The second patient, born in 1992 and of Han nationality, came to our hospital in 2018 due to no pregnancy for 6 years without contraception. She has a history of three abortions, with her last pregnancy in 2012. Despite regular unprotected sexual activity since then, she has not conceived. Hysterosalpingography (HSG) in 2015 at another hospital revealed bilateral tubal obstruction, which was addressed via laparoscopic reconstructive surgery, resulting in patent fallopian tubes. However, a 2016 HSG showed renewed right-sided obstruction, treated again to restore patency in both tubes. Postoperatively, attempts to conceive were unsuccessful. An August 2017 HSG reiterated right-sided obstruction. From September 2018 to October 2021, she underwent four cycles of IVF at our hospital, obtaining 5, 3, 1 and 3 frozen embryos, respectively. Between January 2019 and November 2019, she attempted embryo transfer four times, experiencing one biochemical pregnancy and three implantation failures (Supplementary Tables S3 and S4).

#### 2.2.2 ERA test findings

In December 2019, an ERA test was performed, indicating that the personalized Embryo Transfer should occur 145 ± 3 h after progesterone application (i.e., delayed by one day from the endometrial biopsy time).

#### 2.2.3 Treatment and outcomes

Between February 2020 and April 2021, two transfers were conducted according to the ERA protocol, each transferring one blastocyst, both resulting in biochemical pregnancies. In December 2021, she underwent another pET of two blastocysts, which resulted in a successful pregnancy and full-term delivery of a healthy baby girl (Supplementary Table S4).

In March 2024, the couple planned for a second child and proceeded with a transfer of one blastocyst according to, which did not result in pregnancy. In May 2024, she underwent an IVF cycle again, obtaining four frozen embryos. In July 2024, a pET with one blastocyst was performed according to the ER test report issued in December 2019. She was confirmed to have a clinical pregnancy with a single gestational sac 3 weeks after transfer. Currently, she has successfully delivered a baby boy.Similarly, ER test guided pET could help to achieve a second successful pregnancy almost five years after biopsy. During the 5-year period, her menstrual cycles also remained regular and stable without significant changes.

As for the methodology of the biopsy cycle of this patient, protocol similar to that of the first case was followed. Endometrial tissue was also collected on the day of *P* + 5 in the HRT mock cycle.

#### 2.2.4 Chronic endometritis screening

A hysteroscopy in April 2019, prior to the ERA test, showed a normal uterine cavity. More importantly, before her second pregnancy attempt in June 2024, another hysteroscopy was performed, and the endometrial biopsy result was negative for plasma cells upon CD138 immunohistochemical staining, effectively ruling out chronic endometritis.

## 3 Discussion

Successful embryo implantation relies on a receptive endometrium which occurred during WOI. ERA test is a molecular diagnostic method for accurately predicting the personalized WOI and guiding pET through adjusting the duration of progesterone exposure. The cases presented herein provide compelling evidence for the long-term reliability of ERA test in guiding personalized embryo transfer, even after a significant period following the initial biopsy and subsequent live birth. This finding might bring some implications for clinical practice, particularly for patients with RIF who are considering additional cycles of ART.

Both patients had a history of multiple IVF embryo transfer failures before undergoing ERA test—five failures for the first patient and four for the second. Despite the first patient undergoing three embryo transfers based on the timing of the ERA test, all attempts failed. It was not until the fourth ERA-guided transfer (the ninth transfer overall) that the patient finally succeeded. Although the patient changed the method of progesterone administration, this was not the main factor, as the patient had previously used vaginal progesterone without success. Compared to the sixth, seventh, and eighth transfers, the availability of high-quality embryos was another crucial factor for the patient's success. The second patient also had two biochemical miscarriages after two transfers based on the ERA test result. It was only after the third pET (the seventh transfer overall) that the patient successfully conceived with the transfer of two blastocysts. The clinical utility of ER-guided pET is evident from the improved pregnancy outcomes observed in these two cases who struggled with infertility and unexplained IVF failures for several years. However, the availability of high-quality embryos remained a key factor for successful pregnancy. In addition, young age is another key factor for good endometrial function and pregnancy success (11). The ages of the two patients at the time of the second successful embryo transfer were 36 and 32 years old, respectively, which were still relatively young (below 40 years). The two successful pregnancies achieved by them suggest that the ERA can effectively identify the optimal WOI for embryo transfer, thereby enhancing the implantation potential. Moreover, the first case was an ethnic minority, indicating ERA's applicability for this group of females.

Simón C, *et al*. had pointed out that test results of ERA were reproducible even up to 40 months after the first test (12). However, the clinical utility of test results beyond a three-year timeframe has not been well-documented. Specifically, it remains unclear whether the findings from an initial ER test continue to provide reliable guidance for personalized embryo transfer several years later. The two cases presented here achieved a second pregnancy success adhering to ERA protocol after more than 54 months post-biopsy, far more than the reported intervals, indicating that the ERA test results might have a long-term reproductivity in individual patients. Moreover, we first found that ER test results still worked even after a previous delivery via caesarean section, indicating childbirth might not alter endometrial receptivity gene expression profiles. Clinicians faced with RIF cases can derive reference from the present cases when recommending ER test as part of the management strategy. However, it is essential to acknowledge that the sample sizes in both this study and in Simón C's are limited, and further research involving larger cohorts is necessary to validate these findings across diverse populations. Given the cost and invasiveness associated with ER test biopsies, knowing whether the results remain valid for several years could spare patients unnecessary procedures and reduce costs.

Simón C, et al. previously indicated that endometrial receptivity as determined by ERA test can change after a successful live birth from a frozen embryo transfer (13). Kuniaki Ota et al. shared a case whose WOI results determined by ERA test before and after antibiotics administration to treat CE were different and suggested that the results of the ERA test may vary in the presence of CE (14). Therefore, while the current cases support the stability of ERA test results over time, it is important to consider various factors that could potentially influence reproducibility and where repeat endometrial biopsy for ER test might be warranted.

In summary, the two case reports presented here highlight the long-term reproducibility and clinical utility of the ERA test in guiding personalized embryo transfer. However, given the small sample size of this case series (only 2), further large prospective studies are still needed to refine the integration of ER test into routine ART protocols, ultimately leading to improved reproductive outcomes for patients struggling with RIF.

## Data Availability

The datasets presented in this article are not readily available because of ethical and privacy restrictions. Requests to access the datasets should be directed to the corresponding authors.
