# Supplementary material for: Long-term reproducibility and clinical utility of endometrial receptivity analysis in guiding personalized embryo transfer: case reports of sustained success over four years post-endometrial biopsy
Source: Front Reprod Health. 2026 Feb 6;8:1769800. doi: 10.3389/frph.2026.1769800 (PMC12920444; doi:10.3389/frph.2026.1769800)
Supplement: Supplementary file 1 [file Table1.docx]

| **Table 1 Patient 1 Ovarian Stimulation** | | | | | | | | | | | | | | | | |
| --- | --- | --- | --- | --- | --- | --- | --- | --- | --- | --- | --- | --- | --- | --- | --- | --- |
| **IVF cycle** | **Date** | **Hospital** | **Ovarian Stimulation** | **Menstrual**  **E2(pg/ml)** | **Menstrual**  **P(ng/ml)** | **Menstrual**  **FSH(miu/ml)** | **Menstrual**  **LH(ng/ml)** | **AFC** | **Gonadotropin** | **Gn initial dose (U)** | **Gn days (d)** | **Gn dose (U)** | **Number of oocytes retrieved (n)** | **Fertilization methods** | **2PN**  **(n)** | **Number of embryos(n)** |
| 1 | 2015.8 | Another hospital | Long GnRH agonist | / | / | / | / | / | Gonal-f | 150 | 11 | 1575 | 11 | IVF | 5 | D3 (8II, 8II) |
| 2 | 2016.5 | Another hospital | Long GnRH agonist | / | / | / | / | / | Gonal-f | 225 | 11 | 2475 | 9 | IVF | 6 | D3 (8II ,8II ,7II ,7II ), |
| 3 | 2019.6 | Our hospital | Long GnRH agonist | 51 | 0.5 | 4.89 | 6.43 | 6 | hMG | 225 | 9 | 2025 | 7 | IVF | 6 | D3 (830,Fusion II),  D6 (6B-B) |
| 4 | 2019.12 | Our hospital | GnRH antagonist | 35 | 0.38 | 6.83 | 0.56 | 10 | hMG | 250 | 11 | 2750 | 9 | IVF | 7 | D3 2(840,730-)  D5 (4B+B) |
| 5 | 2020.12 | Our hospital | GnRH antagonist | 44 | 1.02 | 6.94 | 4.52 | 11 | hMG | 225 | 8 | 1800 | 10 | ICSI | 7 | D5  (4BB,4BB, 4B+B+,4AB-,5BB) |

| **Table 2 Patient 1 Embryo transplantation** | | | | | | | | | | | | | | | | |
| --- | --- | --- | --- | --- | --- | --- | --- | --- | --- | --- | --- | --- | --- | --- | --- | --- |
| **Embryo transplantation cycle** | **Date** | **Endometrial preparation protocol** | **Menstrual**  **E2(pg/ml)** | **Menstrual**  **P(ng/ml)** | **Menstrual TVS Endometrial thickness(mm)** | **Endometrial preparation Drug** | **Endometrial preparation time(day)** | **Endometrial thickness(mm)** | **Before use progesterone**  **E2(pg/mL)** | **Before use progesterone**  **P(ng/ml)** | **Progesterone administration** | **Time of progesterone administration(day)** | **Type of embryo transferred** | **Number of embryos(n)** | **Transferred embryo** | **Out come** |
| 1 | 20158 | Fresh embryo transfer | / | / | / | / | / | / | / | / | / | Opu3d | Cleavage stage | 2 | 8II, 8II | No  pregnancy |
| 2 | 2016.5 | Fresh embryo transfer | / | / | / | / | / | / | / | / | / | Opu3d | Cleavage stage | 2 | 8II, 8II | No  pregnancy |
| 3 | 2016.7 | FET(HRT) | / | / | / | / | / | / | / | / | / | 3d | Cleavage stage | 2 | 7II ,7II | No  pregnancy |
| 4 | 2019.4 | FET(HRT) | 133 | 1.08 | 5 | estradiol valerate 3mg po bid | 10 | 8 | 185 | 0.92 | utrogestan vaginal 200 mg capsules tid,dydrogesterone 20 mg po bid | 3d | Cleavage stage | 2 | 830,Fusion II | No  pregnancy |
| 5 | 2019.10 | FET((HRT with GnRH-a) pretreatment ) | 67 | 0.44 | 5 | Femerton® tablets (2mg, po bid) and Estradiol Gel (5 g, externally, bid) | 10 | 8 | 284 | 0.08 | progesterone 60 mg im qd, Femerton®two yellow tablets  po bid, | 5d | Blastocyst | 1 | 6B-B | No  pregnancy |
| ERA | 2020.3 | FET(HRT) | 80 | 0.2 | 3 | Femerton® tablets (2 mg, po bid,) and Estradiol Gel (5 g, externally, bid) | 10 | 8.9 | 424 | 0.17 | progesterone 60 mg im qd, Femerton®two yellow tablets  po bid, | 5d | hysteroscopy examination and ERA test | | | personalized embryo transfer (pET) for day-5/6 Blastocysts should occur at 133 ± 3 hours |
| 6 | 2020.4 | FET(HRT) | 82 | 0.64 | 4.5 | Same ERA regimen | 13 | 9 | 1437 | 0.09 | Same ERA regimen | 134.5hours | Blastocyst | 1 | 4B+B | No  pregnancy |
| 7 | 2020.8 | FET(HRT | 64 | 0.64 | 4.5 | Same ERA regimen | 15 | 9 | 1035 | 0.08 | Same ERA regimen | 86.5hours | Cleavage stage | 1 | 840 | No  pregnancy |
| 8 | 2020.10 | FET((HRT with GnRH-a) pretreatment ) | 50 | 0.18 | 3.5 | Same ERA regimen | 12 | 9 | 506 | 0.08 | Same ERA regimen | 87hours | Cleavage stage | 1 | 730- | No  pregnancy |
| 9 | 2021.3 | FET(HRT) | 69 | 0.83 | 3 | Same ERA regimen | 10 | 8.9 | 617 | 0.08 | utrogestan vaginal 300 mg capsules bid,Femerton®two yellow tablets  po bid, | 130.5hours | Blastocyst | 1 | 5BB | live birth |
| 10 | 2024.9 | FET(HRT) | 53 | 1.02 | 4 | Same ERA regimen | 11 | 9 | 266 | 0.08 | utrogestan vaginal 300 mg capsules bid,Femerton®two yellow tablets  po bid, | 130.5hours时 | Blastocyst | 1 | 6BB | live birth |

| **Table 3 Patient 2 Ovarian Stimulation** | | | | | | | | | | | | | | | | | |
| --- | --- | --- | --- | --- | --- | --- | --- | --- | --- | --- | --- | --- | --- | --- | --- | --- | --- |
| **IVF cycle** | **Time** | **Hospital** | **Ovarian Stimulation** | **Menstrual**  **E2(pg/ml)** | **Menstrual**  **P(ng/ml)** | **Menstrual**  **FSH(miu/ml)** | **Menstrual**  **LH(ng/ml)** | **AFC** | **Gonadotropin** | **Gn initial dose (U)** | **Gn days (d)** | **Gn dose (U)** | **Number of oocytes retrieved (n)** | **Fertilization methods** | **2PN**  **(n)** | **Number of embryos(n)** |  |
| 1 | 2018.10 | Our hospital | Long GnRH agonist | 71 | 0.57 | 7.48 | 5.89 | 20 | hMG | 150 | 12 | 1650 | 16 | IVF | 5 | D3(830) Blastocyst(3C+B,4CC,4CC,4CC) |  |
| 2 | 2019.9 | Our hospital | GnRH antagonist | 37 | 0.85 | 8.84 | 9.11 | 11 | Gonal-f | 187.5 | 8 | 1500 | 9 | IVF | 6 | D3 (730,830),  Blastocyst(4B-B+) |  |
| 3 | 2020.9 | Our hospital | GnRH antagonist | 41 | 0.61 | 10.63 | 9.58 | 15 | hMG | 150 | 9 | 1350 | 15 | Later-ICSI | 6 | Blastocyst(4BB+) |  |
| 4 | 2021.10 | Our hospital | GnRH antagonist | 48 | 0.61 | 8.25 | 6.9 | 24 | hMG | 150 | 8 | 1200 | 14 | ISCI | 8 | Blastocyst  (4B+B+, 4BB+,4BB-) |  |
| 5 | 2024.5 | Our hospital | GnRH antagonist | 62 | 0.21 | 6.07 | 6.40 | 10 | hMG | 150 | 10 | 1500 | 14 | ICSI | 11 | Blastocyst (4B-B-, 4BB-, 4BB-,4B-B-) |  |

| **Table 4 Patient 2 Embryo transplantation** | | | | | | | | | | | | | | | | | |
| --- | --- | --- | --- | --- | --- | --- | --- | --- | --- | --- | --- | --- | --- | --- | --- | --- | --- |
| **Embryo transplantation cycle** | **Time** | **Endometrial preparation protocol** | **Menstrual**  **E2(pg/ml)** | **Menstrual**  **P(ng/ml)** | **Menstrual TVS Endometrial thickness(mm)** | **Endometrial preparation Drug** | **Endometrial preparation time(day)** | **Endometrial thickness(mm)** | **Before use progesterone**  **E2(pg/mL)** | **Before use progesterone**  **P(ng/ml)** | | **Progesterone administration** | **Time of progesterone administration(day)** | **Type of embryo transferred** | **Number of embryos(n)** | **Transferred embryo** | **Out come** |
| 1 | 2019.1 | FET(HRT) | 74 | 1.14 | 3.5 | Estradiol valerate 3mg po bid | 12 | 8 | 198 | 1.13 | | Progesterone 60 mg im qd, dydrogesterone 20 mg po bid | 5d | Blastocyst | 2 | 4CC,3C+B | Biochemical miscarriage |
| 2 | 2019.4 | FET(HRT) | 61 | 0.42 | 3 | Estradiol valerate 3mg po bid | 14 | 9 | 205 | 0.97 | | Utrogestan vaginal 200 mg capsules tid,dydrogesterone 20 mg po bid | 5d | Blastocyst | 2 | 4CC,4CC | No  pregnancy |
| 3 | 2019.6 | FET(HRT) | 38 | 1.61 | 5 | Femerton® tablets (2 mg, po bid,) and Estradiol Gel (5 g, externally, bid) | 10 | 8.5 | 749 | 0.11 | | Utrogestan vaginal 300 mg capsules bid,Femerton®two yellow tablets  po bid, | 3d | Cleavage stage | 1 | 830 | No  pregnancy |
| 4 | 2019.11 | FET(naturecycle) | 34 | 2.33 | 5 | / | 13 | 9.5 | 351 | | 0.92 | Dydrogesterone 20 mg po bid | LH+5d | Cleavage stage | 2 | 730  830 | No  pregnancy |
| ERA | 2019.12 | FET(HRT) | 27 | 1.27 | 4.5 | Femerton® tablets (2 mg, po bid,) and Estradiol Gel (5 g, externally, bid) and  Femerton® tablets (1 mg, pv qd | 15 | 8 | 2288 | 0.08 | | Progesterone 60 mg im qd, Femerton®two yellow tablets  po bid, | 5d | ERA test Personalized embryo transfer (pET) for day-5/6 Blastocysts should occur at 145± 3 hours | | | / |
| 5 | 2020.2 | FET(HRT) | 54 | 0.85 | 5 | Same ERA regimen | 15 | 10.5 | 2321 | 0.13 | | Same ERA regimen | 146.5hours | Blastocyst | 1 | 4B-B+ | Biochemical miscarriage |
| 6 | 2021.4 | FET(HRT) | 44 | 0.51 | 4.5 | Same ERA regimen | 11 | 11 | 101 | 0.11 | | Same ERA regimen | 146.5hours | Blastocyst | 1 | 4BB+ | Biochemical miscarriage |
| 7 | 2021.12 | FET(HRT) | 40 | 0.38 | 3 | Same ERA regimen | 14 | 9 | 2213 | 0.83 | | Same ERA regimen | 146.5hours | Blastocyst | 2 | 4B+B+, 4BB+ | live birth |
| 8 | 2024.3 | FET(HRT) | 45 | 0.58 | 3 | Same ERA regimen | 20 | 7.7 | 1228 | 0.39 | | Same ERA regimen | 146.5hours | Blastocyst | 1 | 4BB- | No  pregnancy |
| 9 | 2024.7 | FET(HRT) | 45 | 0.14 | 4 | Same ERA regimen | 19 | 7.8 | 1753 | 0.08 | | Same ERA regimen | 146.5hours | Blastocyst | 1 | 4BB- | live birth |

**Abbreviated words**

Hormonal replacement treatment(HRT)

frozen-thawed embryo transfer(FET)

gonadotrophin releasing hormoneagonist (GnRHa )

ovum pick-up(opu)
