# Supplementary material for: Long-term reproducibility and clinical utility of endometrial receptivity analysis in guiding personalized embryo transfer: case reports of sustained success over four years post-endometrial biopsy
Source: Front Reprod Health. 2026 Feb 6;8:1769800. doi: 10.3389/frph.2026.1769800 (PMC12920444; doi:10.3389/frph.2026.1769800)
Supplement: Supplementary file 2 [file Datasheet1.pdf]

CARE Checklist of information to include when writing a case report

CARE case report guidelines

(cc) BY-NC-ND

Topic	Item	Checklist item description	Reported on Line
**Title**	**1**	The diagnosis or intervention of primary focus followed by the words "case report"	Line 0-2, Line 10
**Key Words**	**2**	2 to 5 key words that identify diagnoses or interventions in this case report, including "case report"	Line 9-10
**Abstract (no references)**	**3a**	Introduction: What is unique about this case and what does it add to the scientific literature?	Line 12-26
	**3b**	Main symptoms and/or important clinical findings	Line 15-20
	**3c**	The main diagnoses, therapeutic interventions, and outcomes	Line 15-20
	**3d**	Conclusion—What is the main "take-away" lesson(s) from this case?	Line 23-26
**Introduction**	**4**	One or two paragraphs summarizing why this case is unique (**may include** references)	Line 27-45
**Patient Information**	**5a**	De-identified patient specific information	Line 47-58, ,99-110
	**5b**	Primary concerns and symptoms of the patient	Line 60,76, 109-110,120
	**5c**	Medical, family, and psycho-social history including relevant genetic information	Line 47-58,99-110
	**5d**	Relevant past interventions with outcomes	Line 76-81
Clinical Findings	6	Describe significant physical examination (PE) and important clinical findings	Line 50-52,103-107
Timeline	7	Historical and current information from this episode of care organized as a timeline	Line 76,107,109,112,116,120
Diagnostic Assessment	**8a**	Diagnostic testing (such as PE, laboratory testing, imaging, surveys).	Line 59-65,111-114
	**8b**	Diagnostic challenges (such as access to testing, financial, or cultural)	NA
	**8c**	Diagnosis (including other diagnoses considered)	Line 59-65,111-114
	**8d**	Prognosis (such as staging in oncology) where applicable	NA
Therapeutic Intervention	**9a**	Types of therapeutic intervention (such as pharmacologic, surgical, preventive, self-care)	Line 64-65
	**9b**	Administration of therapeutic intervention (such as dosage, strength, duration)	Line 64-65
	**9c**	Changes in therapeutic intervention (with rationale)	Line 60-65,111-114
Follow-up and Outcomes	**10a**	Clinician and patient-assessed outcomes (if available)	NA
	**10b**	Important follow-up diagnostic and other test results	NA
	**10c**	Intervention adherence and tolerability (How was this assessed?)	NA
	**10d**	Adverse and unanticipated events	NA
**Discussion**	**11a**	A scientific discussion of the strengths AND limitations associated with this case report	Line 151,152,158-161
	**11b**	Discussion of the relevant medical literature **with references**	Line 153-174
	**11c**	The scientific rationale for any conclusions (including assessment of possible causes)	Line 158-167
	**11d**	The primary "take-away" lessons of this case report (without references) in a one paragraph conclusion	Line 175-179
**Patient Perspective**	**12**	The patient should share their perspective in one to two paragraphs on the treatment(s) they received	Line 60-61
**Informed Consent**	**13**	Did the patient give informed consent? Please provide if requested	**Yes** ☐ √ **No** ☐
